# Case Report: A rare case of prune belly syndrome with intraperitoneal cryptorchidism

**DOI:** 10.3389/fped.2025.1624556

**Published:** 2025-09-04

**Authors:** Weiwei Ji, Huiling Xue, Ying Gu, Yanmin Wan, Liangsheng Lu, Yong Fan

**Affiliations:** ^1^ Department of Pediatric Urology, Children’s Hospital of Fudan University, Shanghai, China; ^2^ Nursing Department, Children’s Hospital of Fudan University, Shanghai, China

**Keywords:** prune belly syndrome, cryptorchidism, urethral obstruction, Fowler-Stephens orchiopexy, microvascular anastomosis

## Abstract

Prune belly syndrome (PBS), a rare congenital disorder characterized by the absence of abdominal wall musculature and abnormalities in the genitourinary tract, is primarily linked to the urethral obstruction during fetal development. Given the overall rarity of the PBS and its multisystem involvement, there is currently no consensus on the optimal management strategy for PBS patients. This case report elaborated the comprehensive therapeutic outcomes of a patient with PBS, who had previously undergone several surgeries, including vesicoscopic cross-trigonal ureteral reimplantation, and first-stage Fowler-Stephens orchiopexy (FSO) for right cryptorchidism. At the age of seven, the patient underwent one-stage FSO combined with microvascular anastomosis for the left intra-abdominal testis. With appropriate perioperative management, the left testis was successfully survived. Herein, we presented an illustrative case to serve as a treatment reference for PBS patients.

## Introduction

Prune belly syndrome (PBS), a rare congenital multisystem disorder, is defined by the triad of abdominal muscle deficiency, bilateral cryptorchidism, and urinary tract abnormalities (1). Surgical management of intra-abdominal testes typically involves either single-stage or two-stage Fowler-Stephens orchiopexy (FSO) (2). In cases with short spermatic vessels or inadequate collateral circulation, microvascular anastomosis can significantly reduce the risk of testicular ischemia. Given the complexity of intraperitoneal cryptorchidism in PBS patients, multidisciplinary collaboration—encompassing surgery, anesthesia, and perioperative nursing—can enhance treatment outcomes. Herein, we report an unusual case of successful testicular survival following combined therapeutic interventions for PBS.

## Case presentation

A 7-year-old boy was admitted to our hospital in February 2022 with a chief complaint of left intra-abdominal cryptorchidism. The child had been found bilateral hydronephrosis in prenatal examinations and then was diagnosed with PBS after birth, characterized by abdominal wall laxity, urinary system abnormalities and undescended testicles. From 2014 to 2017, the patient received sequential surgical treatments in our hospital, including ureteroplasty, vesicoscopic cross-trigonal ureteral reimplantation, and double-J stent implantation. The child underwent a one-stage FSO for right-sided cryptorchidism at the age of six months. During the procedure, an ultrasonic scalpel was used to cut off the spermatic cord vessels, while collateral vessels connected to the testis were carefully preserved. The testis was then successfully repositioned into the scrotum. Postoperative B-ultrasound revealed diminished blood flow to the right testis, raising suspicion of testicular atrophy. Given the possibility of similar conditions might occur in a one-stage FSO for left cryptorchidism within a short period, we opted to postpone the surgery and wait for the child to mature, as the thickening of blood vessels over time may lead to a better surgical outcome. Thereafter, annual B-ultrasound examinations were performed, showing a gradual restoration of blood supply to the right testis, with no signs of atrophy observed during physical examination. Considering the long-term risk of malignant transformation associated with left abdominal cryptorchidism, the clinical team decided to proceed with one-stage FSO combined with microarteriostomy following a period of observation. During this time, the patient experienced recurrent urinary tract infections (UTIs), which were effectively managed with antibiotic therapy.

**Figure 1:**
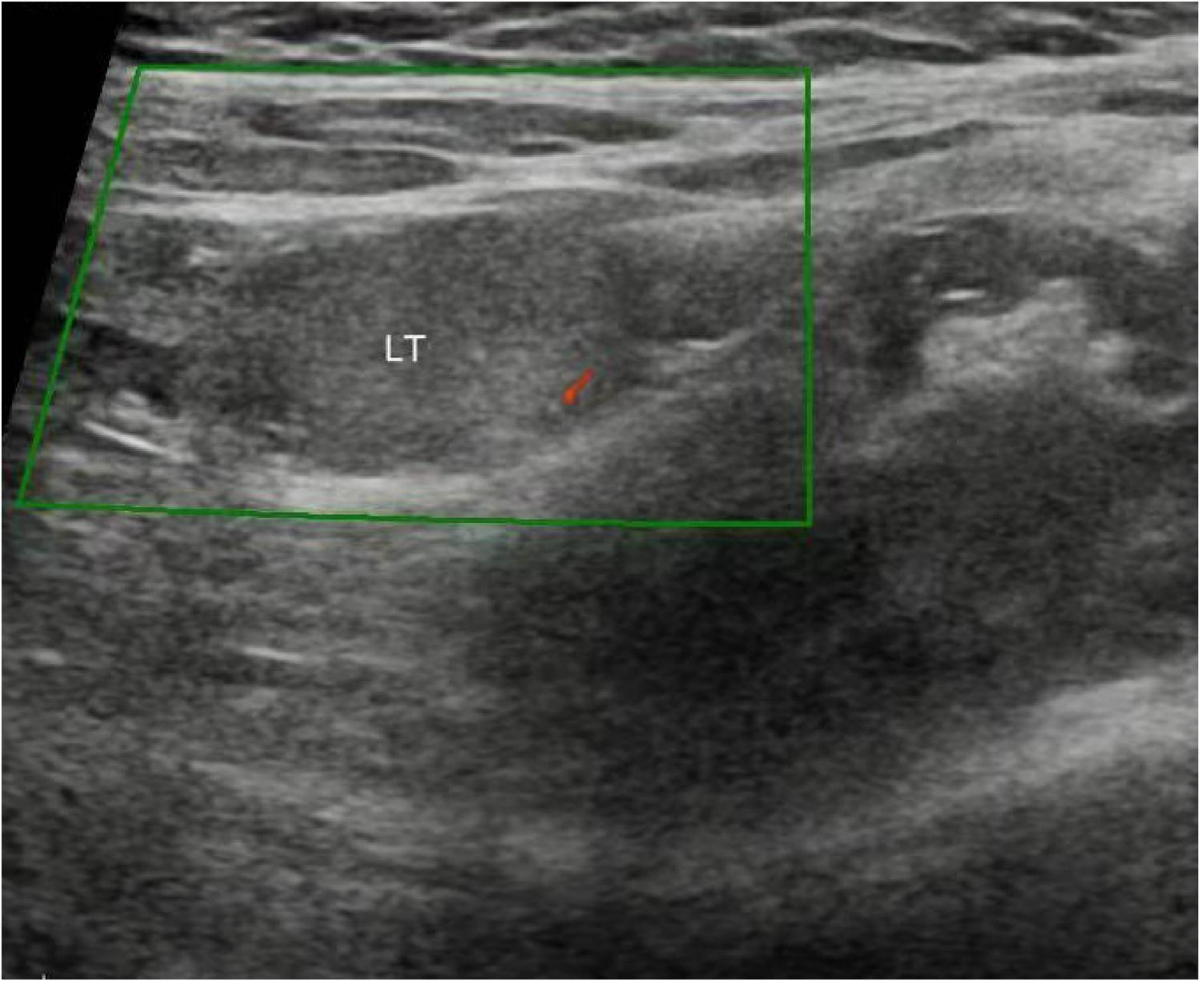
Preoperative B-ultrasound showed that the left testicle was situated in the abdominal cavity (green box).

**Figure 2:**
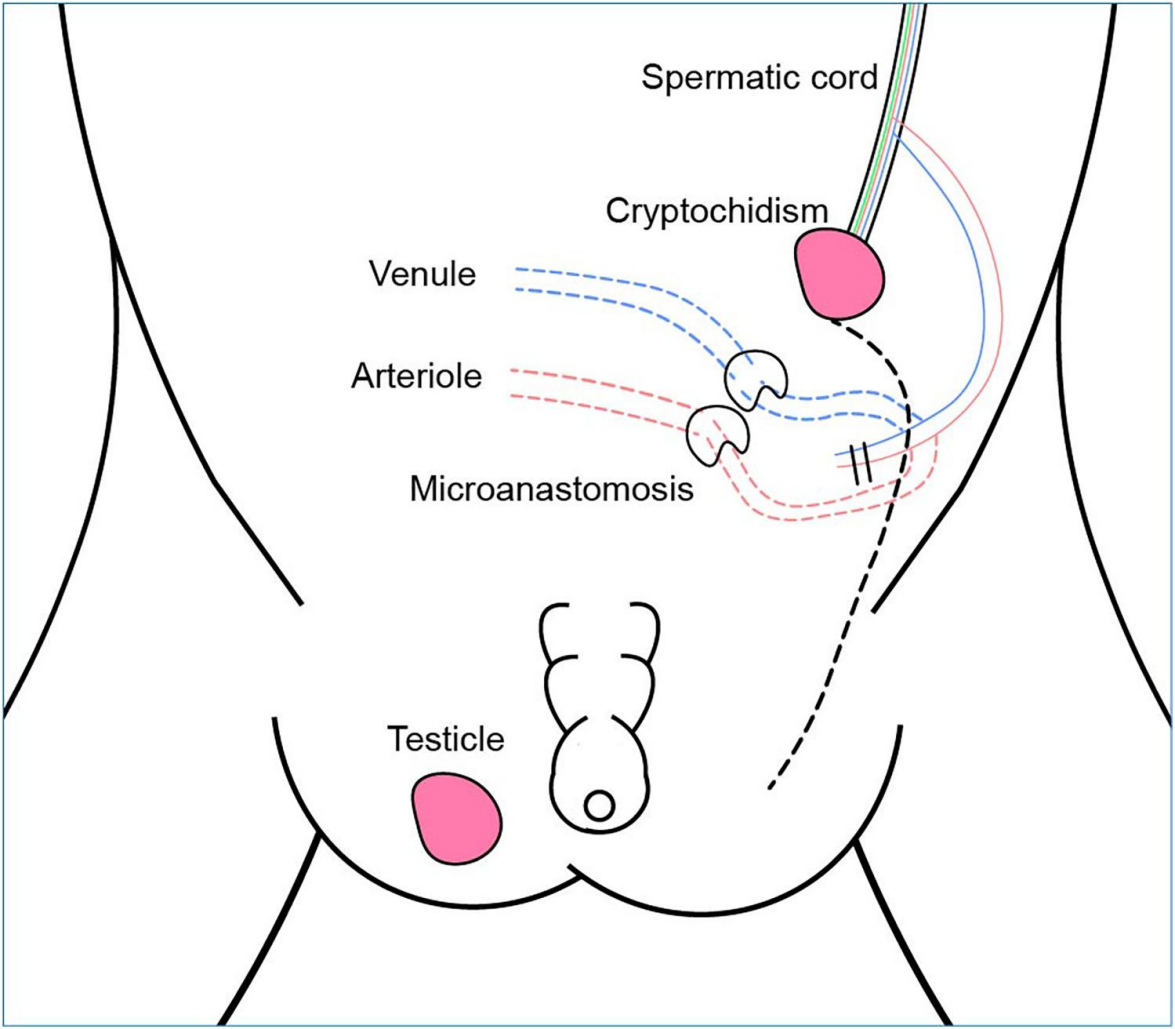
Schematic diagram of one-stage FSO combined with microvascular anastomosis. An additional feeding artery was added to the existing collateral blood supply. The green lines within the spermatic cord are the vas deferens.

**Figure 3:**
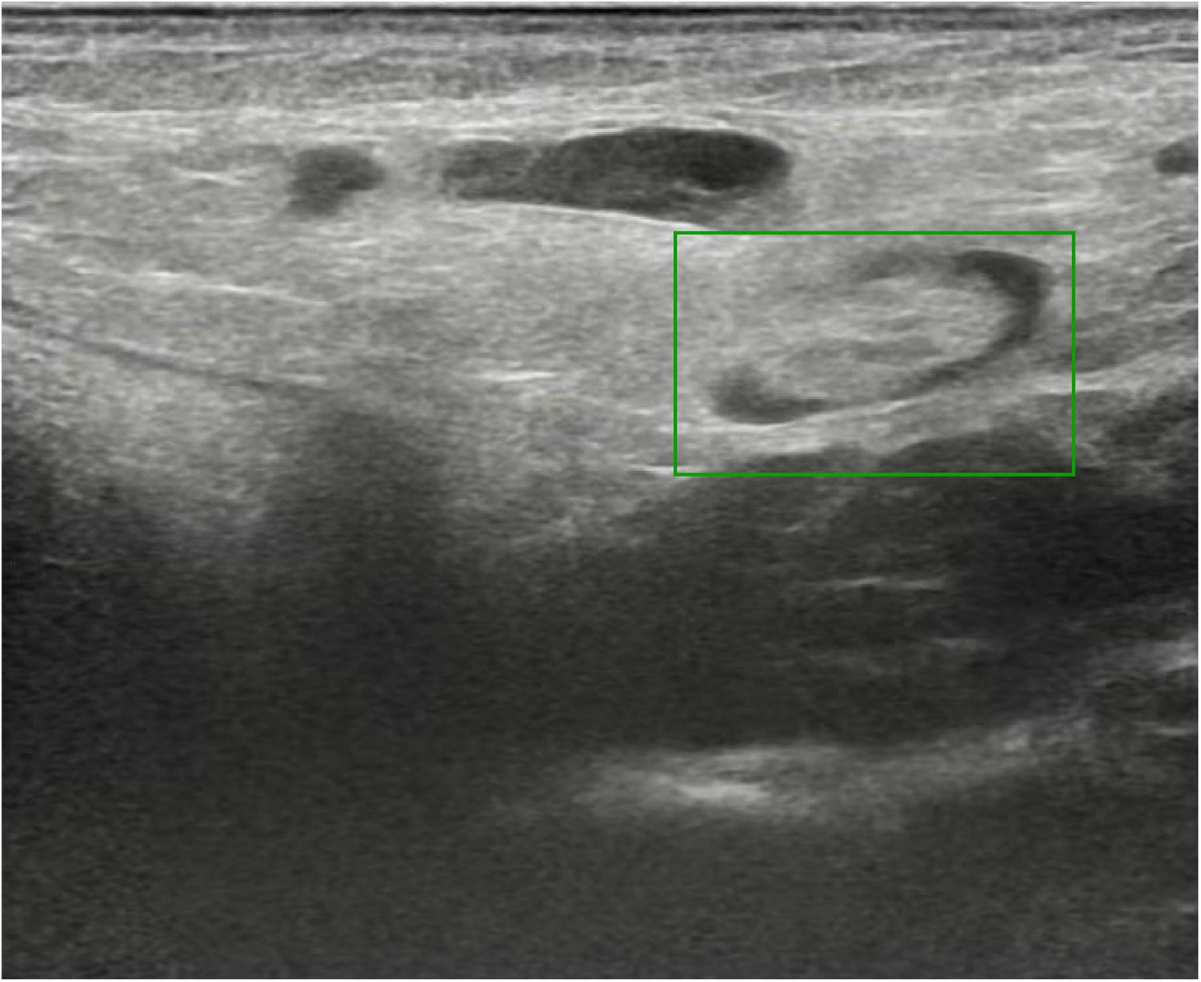
The post-operative B-ultrasound scan indicated that the left testicle was positioned within the scrotum (green box).

The patient was readmitted to the hospital in February 2022 for treatment of left cryptorchidism. B-ultrasound imaging showed that the testicular tissue was located in the left lower pelvic cavity with 14.4 × 9.8 × 8.6 mm in size (Figure 1). Following preoperative and nursing preparations, the patient received laparoscopic surgery under general anesthesia. During the surgery, it was observed that the left testicle was positioned 3 cm above the internal inguinal ring opening. The blood vessels surrounding the vas deferens were not prominent, and the spermatic cord vessels were relatively short, rendering routine orchiopexy for testicular descent unfeasible. According to the preoperative plan, a channel from the abdominal cavity to the scrotum was established. The left testicle was then isolated, and the superficial inferior epigastric arteriole was chosen as the feeding artery, due to the inferior epigastric artery and superficial circumflex iliac artery were deemed too thick. Microanastomosis was performed between the superficial inferior epigastric arteriole and the spermatic artery using interrupted 10-0 nylon and 11-0 nylon sutures to establish a continuous vascular lumen (Figure 2). Then blood vessel patency test was promptly performed to ensure proper blood flow. Subsequently, the vein was anastomosed, after which the testicle were transposed into the scrotum. The entire procedure lasted approximately 2 h. Low molecular weight heparin calcium was administered as an anticoagulant therapy from the day of surgery through the third postoperative day. On the fifth day after surgery, B-ultrasound examination showed good testicular position and texture (Figure 3). Following discharge, multiple follow-up evaluations in the local hospital using B-ultrasound confirmed adequate testicular blood supply in patient. On April 5, 2025, an ultrasound examination (US654263) conducted at Anhui Provincial Children's Hospital revealed color blood flow signals in both testes. The left testis measured 27 × 9 mm, while the right testis measured 18 × 8 mm.

## Discussion

**Figure 4:**
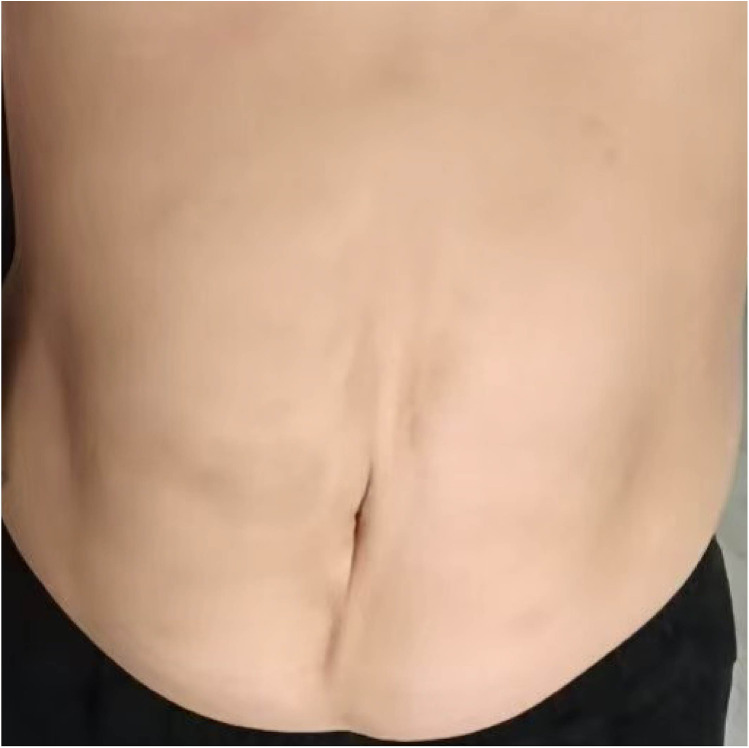
Images of the child patient's anterior abdominal wall dysplasia.

PBS, also known as Eagle-Barrett Syndrome, has an incidence of 3.76 cases per 100,000 live male births (3). Advances in prenatal screening have contributed to a reduced incidence of PBS in developed countries and regions. Most PBS cases occur sporadically and present with normal karyotypes. The absence of effective PBS experimental animal models hinders basic research on this disease. Although several genetic variants associated with PBS have been identified in recent years, the genetic basis for the majority of PBS cases remains unclear (4–6). Primary mesenchymal developmental defects and urethral obstruction are the predominant theories regarding the embryogenesis of PBS. These theories suggest that such defects can lead to dysplasia of the abdominal wall musculature (Figure 4) and the urinary system, as well as prevent testicular descent (7).

The clinical presentation of Prune-Belly syndrome (PBS) is highly heterogeneous, ranging from mild, single-system involvement to life-threatening multi-organ malformations. According to the Woodard classification system, children with PBS can be categorized into three groups: Category I, characterized by pulmonary and renal hypoplasia, typically results in neonatal mortality; Category II includes patients with the classic triad of symptoms and relatively preserved renal function; Category III represents the mildest form, presenting with only abdominal wall defects, cryptorchidism, and minor urinary abnormalities (8). The patients in this study were classified under Category II. The extent of urinary tract involvement, particularly renal dysplasia, is a key factor in determining long-term survival (9). The patient in this report was diagnosed with worse chronic kidney disease (stage 3), along with hydroureteronephrosis and renal hypertension, following a multidisciplinary consultation in the cardiology and nephrology departments of our hospital. Through a combination of medication and dietary management, the patient's blood pressure was effectively controlled. In addition, patients suffer from urinary tract malformations that result in recurrent urinary tract infections, potentially worsening renal function and increasing the frequency of hospital admissions. Urinary tract infections in PBS patients with upper urinary tract dilation have been shown to potentially induce hyperammonemic encephalopathy (10). Following personalized urological interventions, such as regular urine culture testing, ureteroplasty and vesicoscopic cross-trigonal ureteral reimplantation, these issues have been substantially alleviated.

Bilateral intra-abdominal testis is one of the main features of PBS. In addition to genetic factors, the decrease in intra-abdominal pressure due to anatomical changes in the anterior abdominal wall is also one of the reasons for the development of cryptorchidism (11). Undescended testicles are linked to infertility and a higher risk of testicular malignancy (12). Laparoscopic orchidopexy and laparoscopically assisted one or two-stage Fowler-Stephens maneuver are the primary surgical techniques used to correct intra-abdominal cryptorchidism. In one-stage FSO, the spermatic vessels are dissected from the testis while preserving the collateral vasal arteries. In two-stage FSO, orchiopexy is performed several months after ligation of the spermatic vessels, allowing time for the collateral blood supply to undergo compensatory enlargement (13). Studies have shown that the rates of testicular atrophy and retraction are comparable between one-stage and two-stage FSO (14). Staged orchiectomy can fully preserve the blood supply to the testis, but postoperative adhesions may increase the risk of injuring the vas deferens or testicular collateral circulation during subsequent procedures. Therefore, one-stage FSO offers a feasible and effective treatment option for intra-abdominal cryptorchidism. The patient had received one-stage FSO for right cryptorchidism at the age of six months. It was suspected that testicular atrophy had occurred in the right testicle following surgery, although blood supply was eventually restored after several years of observation. To reduce the risk of postoperative atrophy of the left-sided cryptorchidism, and considering the lack of visible blood vessels surrounding the vas deferens and the short spermatic cord vessels, a one-stage FSO combined with microarteriostomy was conducted. Although this approach has been documented in the literature, its adoption remains limited due to technical challenges (15–17). Currently, despite that some reports on laparoscopic orchidopexy in PBS predominantly address technical issues, less focused on testicular survival. In this report, the patient was followed up several years post-treatment, revealing good testicular texture and adequate blood supply. Furthermore, the timing of treatment for cryptorchidism requires careful consideration. Because patients with PBS experience a significant decrease in the number of germinal epithelial cells, orchidopexy should be performed as early as possible to maximize the potential for fertility (1, 18). However, conducting the surgery too early poses technical challenges.

Considering the rarity and complexity of the disease, clinical care for PBS encounters several key challenges. For instance, due to the dysplasia of the abdominal wall, the child often experienced symptoms such as dry stools and constipation. To facilitate the exposure of surgical field during the operation and minimize the risk of intestinal injury, the patient was administered with a warm saline plus glycerin enema in the evening before surgery and the morning of the operation day. Additionally, the critical nursing priorities following vascular microanastomosis are to prevent both bleeding and thrombosis in order to ensure testicular survival (19). Consequently, postoperative hemostatic drugs were avoided. During hospital stay, no instances of wound bleeding or coagulation dysfunction occurred in the patient. At the same time, it is important to provide guidance to PBS patients after discharge.

Our study has several limitations. As PBS is a rare condition, findings from a single case may not be widely applicable. Due to the rarity of PBS, this research is limited to a case report with a small sample size and a relatively short follow-up period. In the future, large-scale, multicenter studies are needed to further validate the advantages of FSO combined with microvascular anastomosis, and to explore the potential of multidisciplinary collaboration in the treatment of PBS, thereby overcoming the limitations of traditional single-department approaches.

## Conclusions

This case report describes a rare instance of testicular survival in PBS patients with high abdominal cryptorchidism following one-stage FSO combined with microvascular anastomosis. This approach proves to be both effective and safe in managing high cryptorchidism. By adding an additional feeding artery to the existing collateral vessels, adequate testicular blood supply is ensured. Although the procedure involves technical complexity, a risk of complications, and challenges in perioperative management, successful outcomes rely heavily on multidisciplinary collaboration. Due to the limited literature and significant phenotypic variability of these malformations, personalized treatment and care are strongly recommended.

## Data Availability

The original contributions presented in the study are included in the article/Supplementary Material, further inquiries can be directed to the corresponding authors.
